# Supplementary material for: Bmi1 marks distinct castration-resistant luminal progenitor cells competent for prostate regeneration and tumour initiation
Source: Nat Commun. 2016 Oct 5;7:12943. doi: 10.1038/ncomms12943 (PMC5059479; doi:10.1038/ncomms12943)
Supplement: Supplementary Information — Supplementary Figures 1-4, Supplementary Tables 1-5 and Supplementary Methods. [file ncomms12943-s1.pdf]

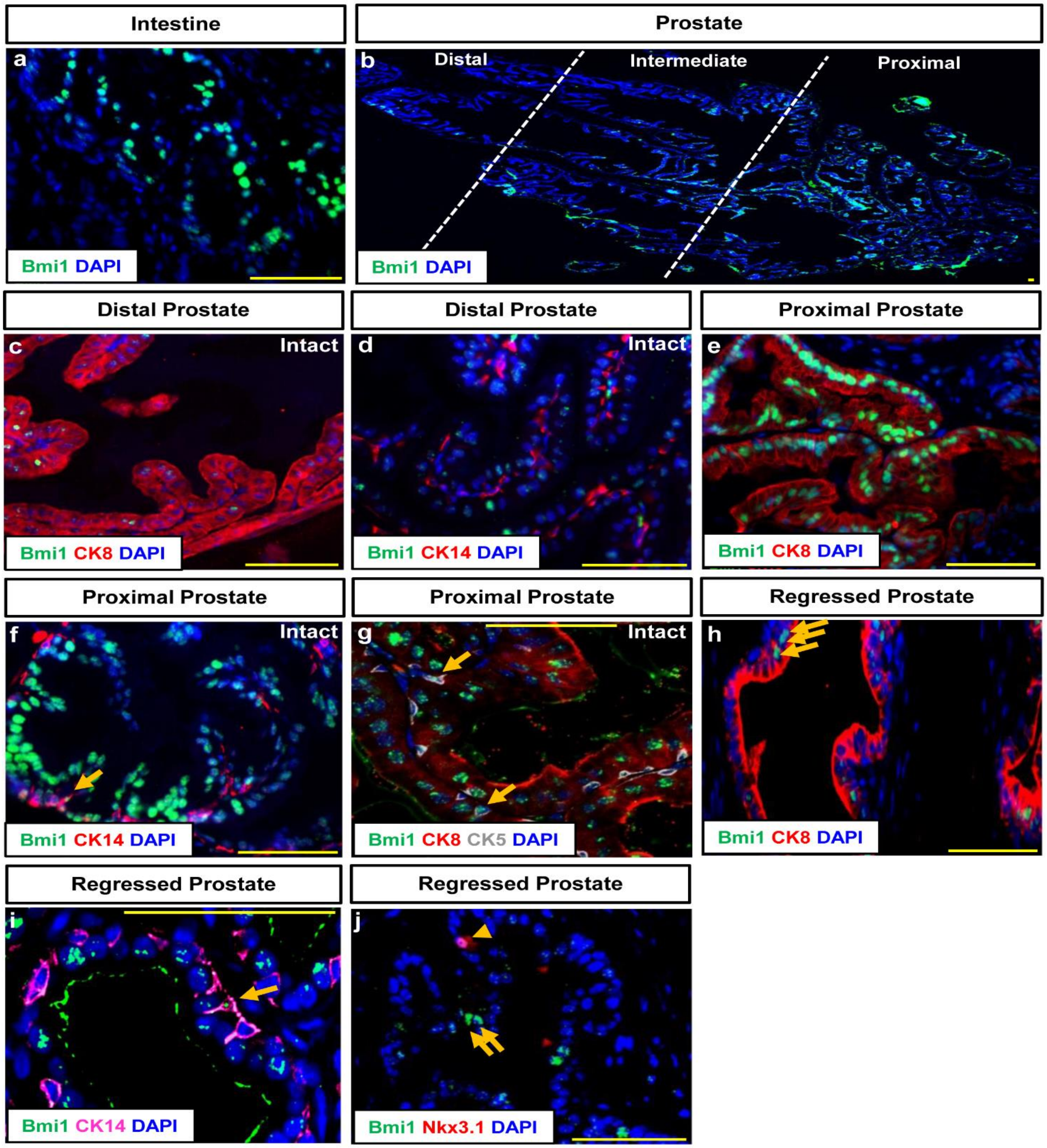

Supplementary Figure 1. Bmi1 protein expression by immunohistochemistry in adult murine prostate. (**a** and **b**) Bmi1 expression (green) in intestinal (a) and prostate epithelium (b). The anterior prostatic glands were divided into proximal, intermediate and distal thirds. (**c** and **d**) Expression and co-localization of Bmi1 with CK8 (**c**) and CK14 (**d**) in distal prostate. (**e-g**) Expression and co-localization of Bmi1 with CK8 (**e**), CK14 (arrow, **f**), or CK5 (arrows, **g**) in proximal prostate. (**h** and **i**) Immunofluorescence staining showing co-localization of Bmi1 with CK8 (arrows, **h**) and CK14 (arrow, **i**) in regressed prostate after 2 weeks of castration. (**j**) Exclusive expression patterns of Bmi1 (arrows) and Nkx3.1 (arrowhead) in the regressed prostate. Scale bars, 50um.

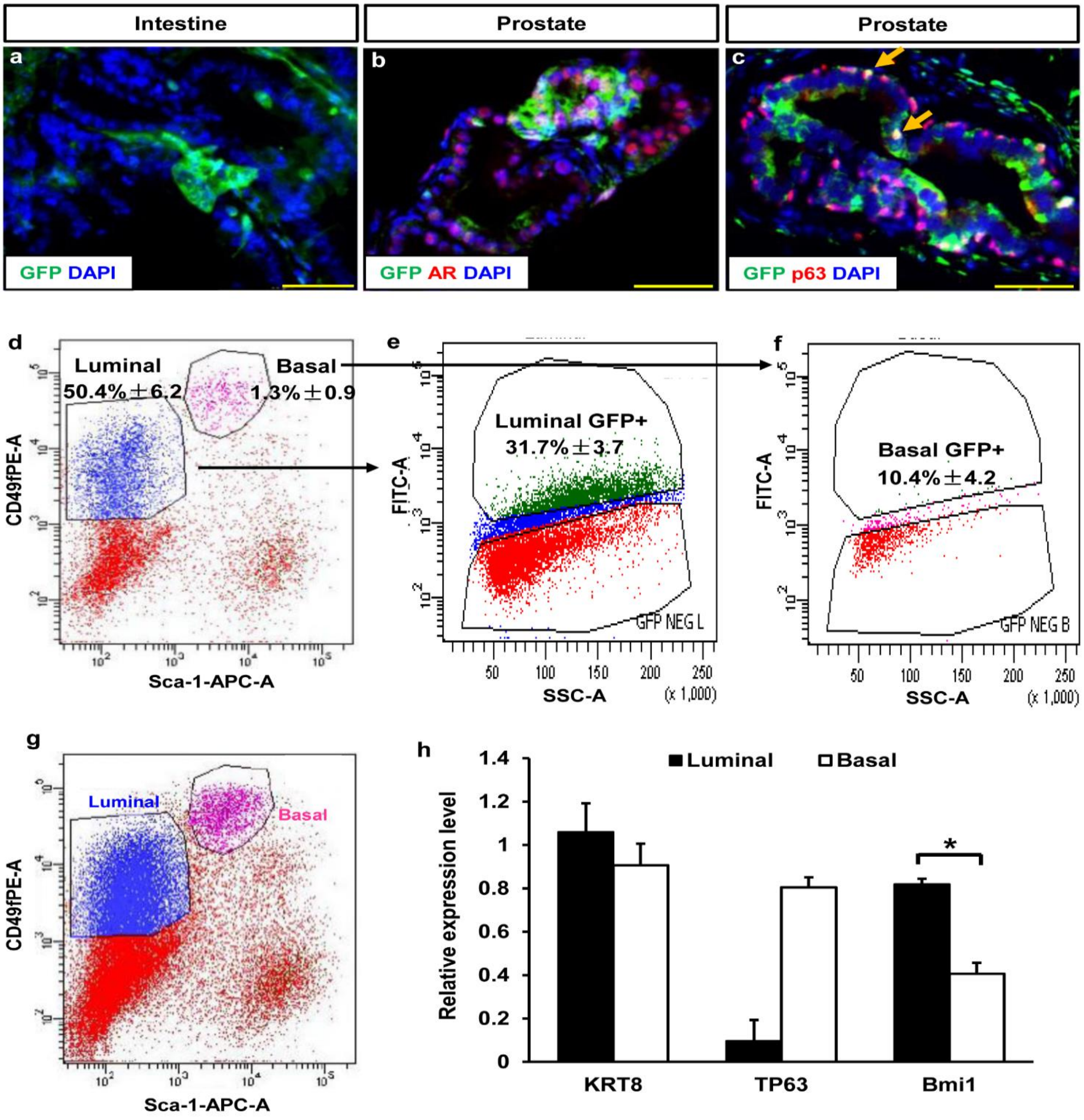

Supplementary Figure 2. GFP expression in the prostate of Bmi1-GFP knock-in mice. (**a-c**) Representative images for GFP expression in the intestinal epithelium (**a**) and co-localization of GFP with AR (**b**) and p63 (arrows, **c**) in the prostate of *Bmi1-GFP* knock-in mice. Scale bar, 50um. (**d**) FACS plot of Lin-Sca1-CD49f^{lo} luminal and Lin-Sca1+CD49f^{hi} basal fractions from the prostate of *Bmi1-GFP* knock-in mice. (e and f) FACS plots showed that corresponding fractions to Lin-Sca1-CD49f^{lo} and Lin-Sca1+CD49f^{hi} each included 31.7% (e) and 10.4% (f) GFP+ cells. (g) FACS plot of Lin-Sca1-CD49f^{lo} luminal and Lin-Sca1+CD49f^{hi} basal fractions from prostate of wild type mice. (h) qRT-PCR analysis of luminal marker *Krt8*, basal marker *Tp63* and *Bmi1* expressions in each FACS-sorted cell fractions. Data represent the mean ± SD from 3 mice. *p<0.05, two-tailed Student's t-test.

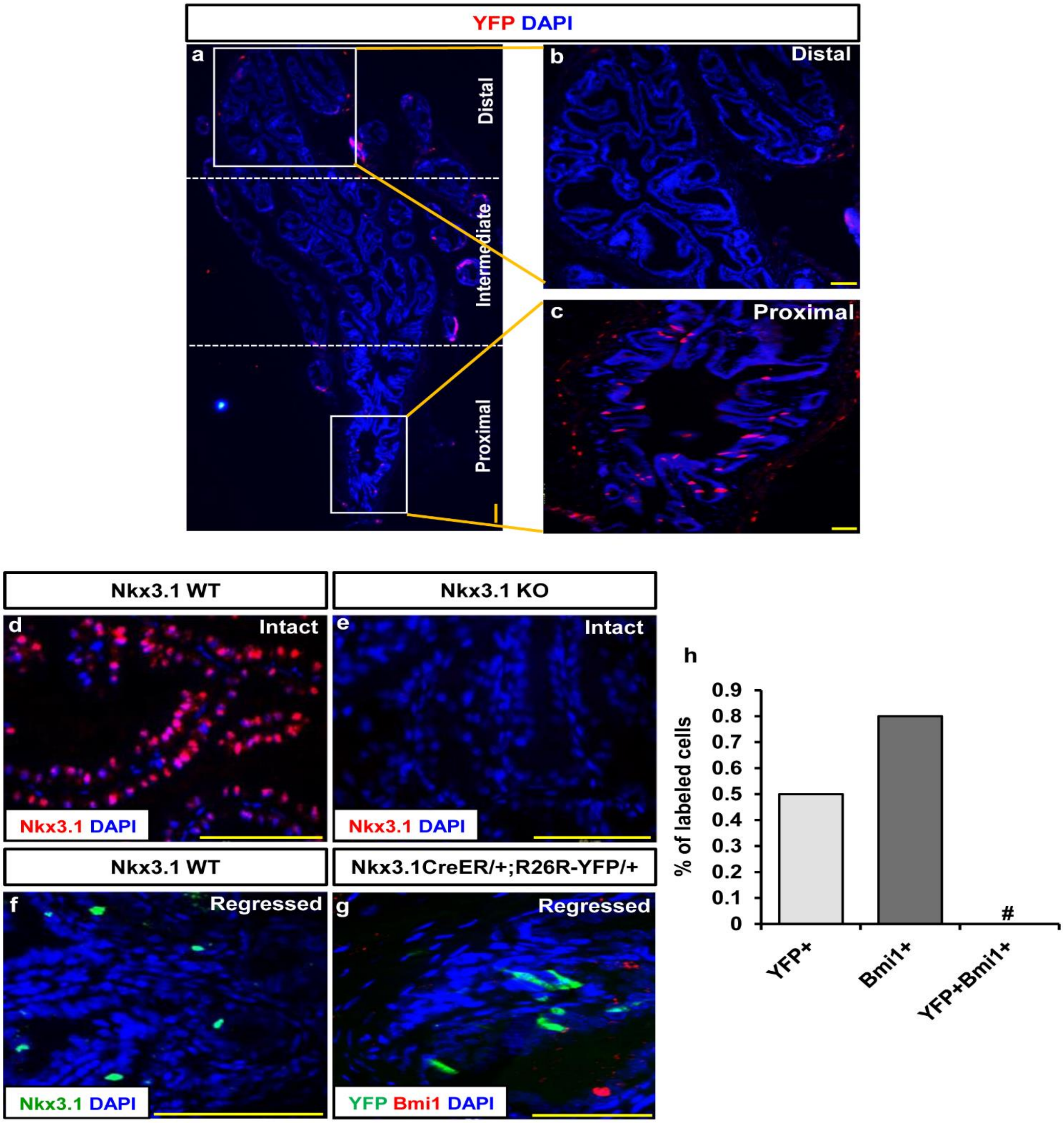

Supplementary Figure 3. Bmi1 mark cells that are distinct from CARNS in the regressed prostate of *Nkx3.1- Cre^{ER};R26R-YFP* mice. (a) Representative image for YFP+ cells that are enrich in the proximal region of anterior prostate. Scale bar, 100um. **(b** and **c)** High magnification image of distal and proximal region, respectively. Scale bar, 50um. (d-f) Representative images for Nkx3.1 expression in the intact prostate of Nkx3.1 wild type (**d**, WT) and knockout (**e**, KO) mutant mice and regressed prostate of Nkx3.1WT mice (**f**). (**g**) Distinct expression patterns of YFP and Bmi1 in the regressed prostate of *Nkx3.1- Cre^{ER};R26R-YFP* mouse. Scale bars, 50um. (**h**) Bar graph shows the quantification of YFP+, Bmi1+, and YFP+Bmi1+ cell fractions in the regressed prostate of *Nkx3.1- Cre^{ER};R26R-YFP* mouse. # No overlap between Bmi1+ cells and CARNs was observed.

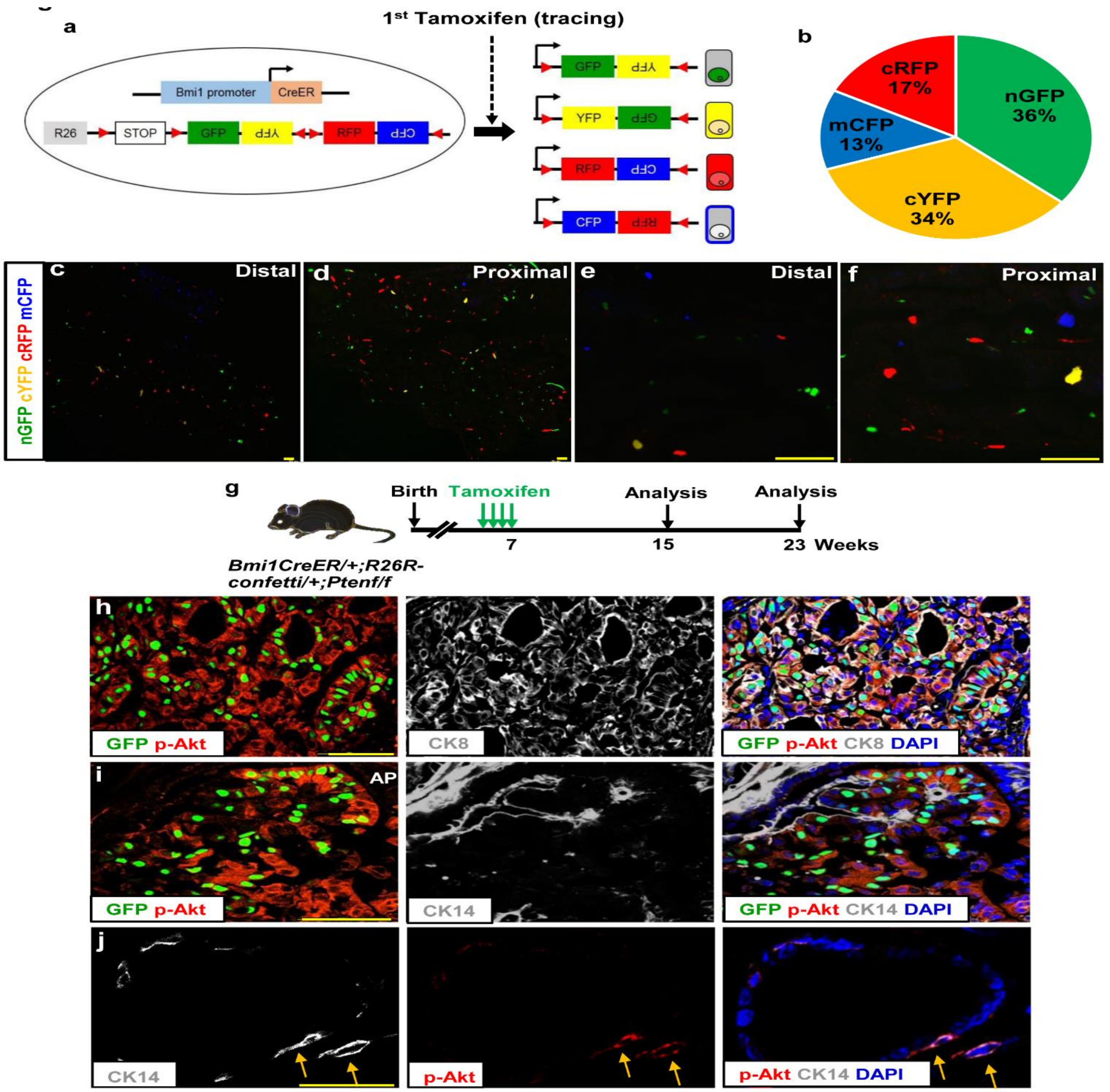

Supplementary Figure 4. Lineage tracing using the *R26R-Confetti* allele. (**a**) Upon induction of Cre recombinase, cells are randomly labelled with one of the four fluorescent proteins (cyan fluorescent protein (CFP), green fluorescent protein (GFP), yellow fluorescent protein (YFP) or red fluorescent protein (RFP)) and grow in a hierarchically organized manner (tracing). (**b**) Quantification of cell fractions labelled with a single *Confetti* color in *Bmi1CreER;R26R-Confetti* mice after 3 or 4 months of tracing (n=164 from 4 mice). (**c-f**) Representative low (c, d) and high (e, f) magnification images for singly labeled confetti cells in both distal (c, e) and proximal (d, f) prostates of intact *Bmi1CreER;R26R-Confetti* mice 1 week post-TAM treatment. (g) *Bmi1CreER;R26R-Confett;Ptenf/f (BC-Pten)* mice were treated with tamoxifen to induce tumor development. (h, i) IF staining shows co-localization of nuclear GFP *Confetti* colors with p-Akt, CK8 (**h**) or CK14 (**i**). (j) IF image showing p-Akt activation due to *Pten* deletion in CK14+ basal cells. Scale bars, 50um.

Supplementary Table 1. Quantitation of Bmi1+ luminal or basal cells in adult prostates

Fraction of Bmi1+ cells out of total luminal and basal cells in AP of intact adult mouse						
Mouse	CK8+	Bmi1+CK8+	Percentage	p63+	Bmi1+p63+	Percentage
1	3324	1861	56.0	2005	559	27.9
2	3481	1941	55.8	1945	405	20.8
3	3351	2347	70.0	1846	362	19.6
4	3556	2156	60.6	2001	422	21.1
5	4004	2268	56.6	1942	360	18.5
Total	17616	10573	60.0	9739	2108	21.6

Fraction of CK8+ vs p63+ cells from total Bmi1+ cells in AP of intact adult mouse						
Mouse	Bmi1+	Bmi1+CK8+	Percentage	Bmi1+	Bmi1+p63+	Percentage
1	1846	1718	93.1	1069	74	6.9
2	1778	1641	92.3	1471	112	7.6
3	2312	2196	95	1399	81	5.8
4	1648	1526	92.6	1048	88	8.4
5	1863	1699	91.2	1173	104	8.9
Total	9447	8780	93	6160	459	7.5

Fraction of CK8+ vs CK14+ or CK5+ cells from total Bmi1+ cells in AP of intact adult mouse							
Mouse	Bmi1+	Bmi1+CK8+	Bmi1+CK14+	Mouse	Bmi1+	Bmi1+CK8+	Bmi1+CK5+
1	3107	3070	38	1	2550	2496	54
2	2520	2448	72	2	2318	2286	32
3	4541	4398	143	3	3742	3647	95
Total	10168	9916 (97.5%)	253 (2.5%)	Total	8610	8429 (97.9%)	181 (2.1%)

Fraction of total Bmi1+ cells in AP of regressed adult mouse			
Mouse	Total epithelial cells	Bmi1+	Percentage
1	7883	128	1.6
2	8665	159	1.8
3	7631	148	1.9
4	8377	164	2.0
5	9257	208	2.2
Total	41813	807	1.9

Fraction of CK8+ vs p63+ cells from total Bmi1+ cells in AP of regressed adult mouse						
Mouse	Bmi1+	Bmi1+CK8+	Percentage	Bmi1+	Bmi1+p63+	Percentage
1	128	119	93.0	291	18	6.2
2	159	147	92.5	313	31	9.9
3	148	139	93.9	289	26	9.0
4	164	155	94.5	267	18	6.7
5	208	193	92.8	368	34	9.2
Total	807	753	93.3	1528	127	8.3

AP: anterior prostate;

Fraction of CK8+ vs CK14+ or CK5+ cells from total Bmi1+ cells in AP of regressed adult mouse							
Mouse	Bmi1+	Bmi1+CK8+	Bmi1+CK14+	Mouse	Bmi1+	Bmi1+CK8+	Bmi1+CK5+
1	52	51	1	#1	44	43	1
2	48	48	0	#2	59	58	1
3	57	55	2	#3	65	63	2
Total	157	154 (98%)	3 (2%)	Total	168	164 (97.6%)	4 (2.4%)

CARNs: castration-resistant Nkx3.1-expressing cells

Supplementary Table 2. Quantitation of lineage-marked luminal, basal, or neuroendocrine cells in intact and regressed prostates of *BY* mice.

**Fraction of total YFP+ cells in AP, VP, and DLP of intact *BY* mouse**									
	AP			VP			DLP		
Mouse	Epithelial cells	YFP+	Percentage	Epithelial cells	YFP+	Percentage	Epithelial cells	YFP+	Percentage
1	16432	73	0.4	5738	23	0.4	8732	31	0.4
2	19536	64	0.3	6811	27	0.4	5854	20	0.3
3	19164	62	0.3	7300	24	0.3	6736	29	0.4
4	17321	52	0.3	6119	19	0.3	6221	21	0.3
5	18435	58	0.3	6843	21	0.3	7049	28	0.4
Total	90888	309	0.3	32811	114	0.3	34592	129	0.4

**Fraction of Bmi1+ cells that were labeled with YFP in AP of intact adult mouse**						
	Distal			Proximal		
Mouse	Bmi1+	YFP+ Bmi1+	Percentage	Bmi1+	YFP+ Bmi1+	Percentage
1	1215	4	0.3	7559	89	1.2
2	1086	3	0.3	5181	67	1.3
3	1266	2	0.2	5624	61	1.1
Total	3567	9	0.3	18364	217	1.2

**Fraction of YFP+ luminal, basal, or neuroendocrine cells in AP of intact adult *BY* mouse**								
		Basal YFP+ cells			Luminal YFP+ cells		Neuroendocrine YFP+ cells	Non-quiescent YFP+ cells
Mouse	YFP+	YFP+ CK14+	YFP+CK5+	YFP+ p63+	YFP+ CK8+	YFP+ AR+	YFP+Syn+	YFP+Ki67+
1	73	2	4	3	69	69	0	0
2	64	3	3	2	61	61	0	0
3	62	2	2	1	60	60	0	0
Total	199	7 (3.5%)	9 (4.5%)	6 (3%)	190 (95.5%)	190 (95.5%)	0 (0%)	0 (0%)

**Fraction of YFP+ luminal, basal, or neuroendocrine cells in AP of regressed adult *BY* mouse**								
	Epithelial cells (n=40662)	Basal YFP+ cells			Luminal YFP+ cells		Neuroendocrine YFP+ cells	Non-quiescent YFP+ cells
Mouse	YFP+	YFP+ CK14+	YFP+CK5+	YFP+ p63+	YFP+ CK8+	YFP+ AR+	YFP+Syn+	YFP+Ki67+
1	147	5	6	3	141	139	0	0
2	59	1	2	2	57	57	0	0
3	100	4	5	3	95	95	0	0
Total	306 (0.7%)	10 (3.3%)	13 (4.2%)	8 (3%)	293 (95.8%)	291 (95%)	0 (0%)	0 (0%)

Supplementary Table 2-continued

**Fraction of overlapping cells between CARBs and CARNs in regressed adult *BY* mouse**			
Mouse	YFP+	Nkx3.1+	YFP+Nkx3.1+ (%)
1	88	386	0
2	63	258	0
3	55	343	0
4	52	352	0
5	64	289	0
Total	322	1628	0

BY: *Bmi1-CreER;R26R-YFP*; AP: anterior prostate; VP: ventral prostate; DLP: dorsolateral prostate; Syn: Synaptophysin; CARBs: castration-resistant Bmi1-expressing cells; CARNs: castration-resistant Nkx3.1-expressing cells

Supplementary Table 3. Quantitation of lineage-marked luminal, basal, or neuroendocrine cells in regenerated anterior prostates of *BY* mice.

**Fraction of YFP+ cells in anterior prostates after 1 round of regression-regeneration**								
	Epithelial cells (n=22748)	Basal YFP+ cells			Luminal YFP+ cells		Neuroendocrine YFP+ cells	Non-quiescent YFP+ cells
Mouse	YFP+	YFP+ CK14+	YFP+CK5+	YFP+p63+	YFP+ CK8+	YFP+ AR+	YFP+Syn+	YFP+Ki67+
1	216	12	18	10	198	196	0	0
2	282	12	9	9	270	264	0	0
3	138	4	5	4	130	130	0	0
Total	636 (2.8%)	32 (5%)	32 (5%)	23 (3.6%)	598 (94%)	590 (93%)	0 (0%)	0 (0%)
**Fraction of YFP cells in 1 round regressed anterior prostates**								
	Epithelial cells (n=19578)	Basal YFP+ cells			Luminal YFP+ cells		Neuroendocrine YFP+ cells	Non-quiescent YFP+ cells
Mouse	YFP+	YFP+ CK14+	YFP+CK5+	YFP+p63+	YFP+ CK8+	YFP+ AR+	YFP+Syn+	YFP+Ki67+
1	34	1	1	1	33	32	0	0
2	37	1	1	0	36	35	0	0
3	38	1	2	2	35	35	0	0
Total	109 (0.6%)	3 (2.8%)	4 (3.7%)	23 (2.8%)	104 (95%)	102 (94%)	0 (0%)	0 (0%)
**Fraction of YFP+ cells in anterior prostates after 2 round of regression-regeneration**								
	Epithelial cells (n=32307)	Basal YFP+ cells			Luminal YFP+ cells		Neuroendocrine YFP+ cells	Non-quiescent YFP+ cells
Mouse	YFP+	YFP+ CK14+	YFP+CK5+	YFP+p63+	YFP+ CK8+	YFP+ AR+	YFP+Syn+	YFP+Ki67+
1	327	11	19	13	308	308	0	0
2	148	8	12	8	136	136	0	0
3	261	12	18	15	243	243	0	0
4	248	15	13	15	233	233		
Total	984 (3.04%)	46 (5%)	62 (6.3%)	51 (5.2%)	920 (94%)	920 (94%)	0 (0%)	0 (0%)
**Fraction of YFP+ cells in anterior prostates after 3 round of regression-regeneration**								
	Epithelial cells (n=24032)	Basal YFP+ cells			Luminal YFP+ cells		Neuroendocrine YFP+ cells	Non-quiescent YFP+ cells
Mouse	YFP+	YFP+ CK14+	YFP+CK5+	YFP+p63+	YFP+ CK8+	YFP+ AR+	YFP+Syn+	YFP+Ki67+
1	198	11	13	11	187	186	0	0
2	257	13	17	14	240	237	0	0
3	283	9	10	7	273	270	0	0
Total	738 (3.1%)	33 (4.5%)	32 (4.3%)	32 (4.3%)	700 (95%)	693 (94%)	0 (0%)	0 (0%)

Supplementary Table 3-continued

Fraction of BrdU incorporated luminal and basal cells by 3 days after regeneration						
Mouse	p63+	63+BrdU+	Percentage	AR+	AR+BrdU+	Percentage
1	2752	47	1.70%	4568	1145	14.00%
2	8853	253	2.90%	11842	3366	28.40%
3	1831	28	1.60%	8727	1909	22.00%
4	2430	41	1.70%	9463	2250	24.00%
Total	15866	369	2.30%	34600	8670	25.00%
**Fraction of BrdU incorporated luminal and basal cells by 7 days after regeneration**						
Mouse	p63+	63+BrdU+	Percentage	AR+	AR+BrdU+	Percentage
1	2106	7	0.30%	4298	101	2.30%
2	5622	51	0.90%	16866	306	1.80%
3	9277	61	0.70%	12428	452	3.60%
4	10521	68	0.60%	13357	294	2.20%
Total	27526	180	0.60%	46949	1153	2.50%

YFP+BrdU incorporated cells by 3 days after regeneration				YFP+BrdU incorporated cells by 7 days after regeneration			
	YFP+ (Total : 956)				YFP+ (Total : 915)		
Mouse	YFP+BrdU+	YFP+ BrdU+p63+	YFP+ BrdU+AR+	Mouse	YFP+BrdU+	YFP+ BrdU+p63+	YFP+ BrdU+AR+
1	32	2	30	1	0	0	0
2	15	0	15	2	3	1	2
3	29	1	28	3	5	0	5
4	20	0	20	4	4	0	4
Total	96 (10%)	3 (3%)	93 (97%)	Total	12 (1.3%)	1 (8.3%)	11 (91.7%)

BrdU incorporation by 3 days after regeneration						
	Total YFP+ cells					
	YFP+ luminal cells			YFP+ basal cells		
Mouse	AR	YFP+AR+	Percentage	p63+	YFP+p63+	Percentage
1	4865	52	1.1%	1535	5	0.3%
2	4184	64	1.5%	1486	6	0.4%
3	5955	59	1.0%	1507	4	0.3%
4	4471	66	1.5%	1614	6	0.4%
Total	19475	241	1.2%	6142	21	0.3%
	Dividing YFP+ cells					
	Dividing YFP+ luminal cells			Dividing YFP+ basal cells		
Mouse	AR+BrdU+	AR+BrdU+ YFP+	Percentage	p63+BrdU+	p63+BrdU+ YFP+	Percentage
1	1373	18	1.3%	52	0	0.0%
2	1295	16	1.2%	48	0	0.0%
3	1428	19	1.3%	56	1	1.8%
4	1357	20	1.5%	40	0	0.0%
Total	5453	73	1.3%	196	1	0.5%

Supplementary Table 4. Primary antibodies

Antigen	Supplier	Species	Dilution
Androgen Receptor	Santa Cruz, N-20	Rabbit	1:200
Bmi1	Cell signaling, D20B7	Rabbit	1:1000
Bmi1	Abcam, #ab 14389	Mouse	1:100
BrdU	Abcam, BU1/75 (ICR1)	Rat	1:100
CK5	Covance, # PRB-160P	Rabbit	1:500
CK8	Covance, # MMS-162P	Mouse	1:500
CK14	Covance, # PRB-155P	Rabbit	1:500
CK14	BioGenex #MU146-UC	Mouse	1:100
GFP	Abcam, #ab13970	Chicken	1:500
Ki67	eBioscience, SolA15	Rat	1:200
Nkx3.1	Novus Biologicals, NB100-1828	Mouse	1:100
Nkx3.1	Athena Enzyme Systems, #0315	Rabbit	1:3000
p63	Santa Cruz, #sc-8343	Rabbit	1:50
phospho-Akt	Cell signaling, #4060	Rabbit	1:50
Synaptophysin	Zymed, #18-0130	Rabbit	1:500
CD49f-PE	eBioscience, #12-0495-83		1:333
Sca1-APC	eBioscience, #17-5981-82		1:500
CD31-PE-CY7	eBioscience, #25-0311-81		1:250
CD45-PECy7	eBioscience, #25-0451-81		1:250
Ter119-PECy7	eBioscience, #25-5921-81		1:250

Supplementary Table 5. Primers

Primer	Sequences (5' – 3')
TP63 Forward	CTTCCGTGAGCCAGCTTATC
TP63 Reverse	GAGTGGAGGAGGGAGAGCTT
KRT8 Forward	AGCTGAGGCTGAAACCATGT
KRT8 Reverse	TTGATGTTGCGGTTCATCTC
36B4 Forward	AGATGCAGCAGATCCGCA
36B4 Reverse	GTTCTTGCCCATCAGCACC
Bmi1	SABiosciences PPM37679E-200

Supplementary Methods

Flow Cytometry Cell Sorting

Prostate glands were digested with collagenase (Invitrogen) in DMEM media with 10% FBS for 2 hours at 37°C. Subsequently, digested cells were suspended in DNase I (Sigma), and then passed through 40-µm cell strainer (Corning Inc., Corning, NY) to obtain single cells. Flow Cytometry Cell Sorting was performed on a BD FACSAria SORP system in the Flow Cytometry Core Facility of Robert H. Lurie Comprehensive Cancer Center. Antibodies used for cell sorting and analysis are listed in Supplementary Table 4.

qRT-PCR

Total RNA extraction was performed using the TRIzol reagent (Life Technologies, Rockville, MD), according to the manufacturer's instructions. Amplification of transcripts was performed using 1 µg/µl of total RNA and the reverse transcriptase polymerase chain reaction (RT-PCR) using Molony murine leukemia virus reverse transcriptase (MMLV; Invitrogen, Carlsbad, CA) and oligo-d(T)16 primer (Invitrogen). qRT-PCR was performed using iTaqTM Universal SYBR Green Supermix kit (BioRad). Primers used for qRT-PCR are listed in Supplementary Table 5.
